# Anti-citrullinated protein antibodies contribute to platelet activation in rheumatoid arthritis

**DOI:** 10.1186/s13075-015-0665-7

**Published:** 2015-08-24

**Authors:** Kim L.L. Habets, Leendert A. Trouw, E.W. Nivine Levarht, Suzanne J.A. Korporaal, Petra A.M. Habets, Philip de Groot, Tom W.J. Huizinga, René E.M. Toes

**Affiliations:** Department of Rheumatology, Leiden University Medical Centre, C1-R, PO Box 9600, 2300 RC Leiden, the Netherlands; Department of Clinical Chemistry and Haematology, University Medical Centre, Utrecht, the Netherlands; Division of Biopharmaceutics, Leiden Amsterdam Centre for Drug Research, University of Leiden, Leiden, the Netherlands; Knowledge Centre Forensic Psychiatric Care, Rekem Psychiatric Hospital, Rekem, Belgium

## Abstract

**Introduction:**

Although the role of platelets in rheumatoid arthritis (RA) is relatively unexplored, recent studies point towards a contribution of platelets in arthritis. We set out to determine platelet phenotype in RA and studied whether this could be influenced by the presence of anti-citrullinated protein antibodies (ACPA).

**Methods:**

Platelets from healthy controls were incubated in the presence of plasma of patients with RA or age- and sex-matched healthy controls and plasma from ACPA^neg^ or ACPA^pos^ patients or in the presence of plate-bound ACPA. Characteristics of platelets isolated from patients with RA were correlated to disease activity.

**Results:**

Platelets isolated from healthy controls displayed markers of platelet activation in the presence of plasma derived from RA patients, as determined by P-selectin expression, formation of aggregates and secretion of soluble CD40 ligand (sCD40L). Furthermore, levels of P-selectin expression and sCD40L release correlated with high ACPA titres. In accordance with these findings, enhanced platelet activation was observed after incubation with ACPA^pos^ plasma versus ACPA^neg^ plasma. Pre-incubation of platelets with blocking antibodies directed against low-affinity immunoglobulin G receptor (FcγRIIa) completely inhibited the ACPA-mediated activation. In addition, expression of P-selectin measured as number of platelets correlated with Disease Activity Score in 44 joints, C-reactive protein level, ACPA status and ACPA level.

**Conclusions:**

We show for the first time that ACPA can mediate an FcγRIIa-dependent activation of platelets. As ACPA can be detected several years before RA disease onset and activated platelets contribute to vascular permeability, these data implicate a possible role for ACPA-mediated activation of platelets in arthritis onset.

**Electronic supplementary material:**

The online version of this article (doi:10.1186/s13075-015-0665-7) contains supplementary material, which is available to authorized users.

## Introduction

Most studies addressing the contribution of the immune system in rheumatoid arthritis (RA) have been focused on white blood cells and antibodies, but the potential role of platelets has received little attention. Although our classical perception of platelets is that of key players in haemostasis and thrombosis, several lines of evidence suggest that platelets also mediate inflammatory processes [1–3]. Platelets carry various mediators facilitating the recruitment of circulating monocytes and/or leucocytes to the injured endothelium and have the potential to propagate vascular permeability and chronic inflammation of the vessel wall [4–6]. Activated platelets are characterised by surface expression of P-selectin (CD62P) and produce both membrane-bound and soluble CD40 ligand (sCD40L). Platelet-derived sCD40L acts as a trigger for the activation and inflammation of the endothelium as it increases the expression of inflammatory adhesion receptors (vascular cell adhesion molecule 1 and intracellular adhesion molecule 1), the production of chemokines (monocyte chemoattractant protein 1, interleukin [IL]-6 and IL-8) and the production of matrix metalloproteinase 9 [7, 8].

RA is characterised by inflammation with progressive destruction of the synovial joints and physical disability [9]. A complex network of small blood vessels is present beneath the surface of the synovium, and enhanced synovial vascularity and biomarkers of angiogenesis have been described in different chronic arthritic diseases [10]. Initially, platelet activation within the joints has been described in mice where platelet depletion resulted in reduced vascular leakage in arthritic joints and attenuated inflammatory arthritis [4, 11, 12]. More recently, it became apparent that such mechanisms might also be present in patients with RA, as increased numbers of platelets and platelet-derived proteins within the synovium and synovial fluid have been observed [11, 13–15]. Furthermore, enhanced levels of soluble P-selectin and sCD40L present in serum and/or plasma correlate with RA activity and suggest potential platelet activity in vivo [16–19]. Importantly, the risk of deep vein thrombosis and pulmonary embolism is increased in patients with RA [20, 21]. However, the mechanism contributing to the putative elevated platelet activity in RA is not known. Because platelets express the low-affinity immunoglobulin G (IgG) receptor (FcγRIIa), and because of the importance of autoantibodies on the thrombotic risk in systemic lupus erythematosus, we propose a role for anti-citrullinated protein antibodies (ACPA) in the activation of platelets [22]. These autoantibodies recognise a group of autoantigens which are post-translationally modified by peptidyl arginine deaminase (PAD) enzymes, leading to the conversion of an arginine to citrulline. ACPA are highly specific for RA and recognise various citrullinated antigens, such as fibrinogen, vimentin, collagen type II and enolase. Typically, 50–70 % of patients with RA are ACPA^pos^, and, of more importance, ACPA can be observed several years before the onset of disease. The presence of ACPA is predictive of a more severe disease course, which necessitates a more aggressive treatment regimen [23–25]. Furthermore, patients with RA have an increased cardiovascular risk, owing mainly to atherosclerotic lesion formation. Intriguingly, the augmented cardiovascular burden in patients with RA is independent of the presence of traditional cardiovascular risk factors such as dyslipidaemia, hypertension, smoking and physical inactivity, but it is associated with the presence of rheumatoid factor (RF)-IgM and ACPA [26–34]. In the present study, we show for the first time an activating interplay between platelets and ACPA. Because of the prominent role of platelets during endothelial dysfunction and vascular permeability that may lead to enhanced vascular leakage in the joints, this interplay could potentially contribute to the increased cardiovascular risk and RA disease burden observed in ACPA^pos^ patients.

## Methods

### Patient characteristics

Samples were obtained from patients with RA (both ACPA^pos^ and ACPA^neg^) who visited the outpatient clinic of the Department of Rheumatology at Leiden University Medical Centre, Leiden, the Netherlands. Patients fulfilled the 1987 criteria for RA [35] at the time of diagnosis and gave us their written informed consent for sample acquisition. Treatment included a wide variety of disease-modifying antirheumatic drugs (DMARDs), biologic agents and glucocorticoids. Peripheral blood for platelet isolation was collected from 32 patients with RA (Disease Activity Score in 44 joints [DAS44] 2.1±0.2). Of these patients, 78 % were ACPA^pos^ (median ACPA 515 AU/ml, interquartile range [IQR] 267.5–2013). For the experiments with plasma samples, healthy platelets were incubated in the presence of plasma of patients with RA. To ensure active disease, only plasma samples from patients with DAS44 > 2.4 were included (DAS44 2.7±0.05). In the first cohort, plasma samples from age-matched (62.4±2.1 vs 61.2±0.8) and sex-matched healthy volunteers were used as a control. In the second cohort, plasma from ACPA^neg^ (ACPA 8.8±1.6 AU/ml) and ACPA^pos^ (ACPA 664.6±137.9) patients with RA was used, and samples were matched for age (56.0±2.8 vs 55.6±2.6), sex and DAS44 score (3.1±0.8 vs 3.24±0.1). Permission for conduct of the study was obtained from the ethical review board of Leiden University Medical Centre. The characteristics of plasma cohorts 1 and 2 are depicted in Table 1.

**Table 1:** Characteristics of plasma cohorts 1 and 2

Plasma cohort 1	Healthy subjects	Patients with RA	*P* value
Female, n (%)	33 (78.6)	36 (81.8)	ns
Male, n (%)	9 (21.4)	8 (18.2)	ns
Age, yr (mean ± SEM)	60.9±0.8	61.83±2.1	ns
TNF-α, pg/ml (median [IQR])	75.0 [51.4–88.9]	87.8 [57.8–126.4]	0.0235
PAF, ng/ml (mean ± SEM)	27.9±1.4	33.4±1.7	0.0185
Plasma cohort 2	ACPA^neg^ patients with RA	ACPA^pos^ patients with RA	*P* value
Female, n (%)	26 (78.8)	25 (75.8)	ns
Male, n (%)	7 (21.2)	8 (24.2)	ns
Age, yr (mean ± SEM)	56.0±2.8	55.6±2.6	ns
DAS44 (mean ± SEM)	3.1±0.8	3.2±0.1	ns
ACPA (AU/ml) (median [IQR])	6.2 [2.6–14.6]	238.7 [98.4–1093]	0.0001
RF IgM (median [IQR])	1.0 [0.0–2.5]	23.0 [6.0–80.0]	0.0001
TNF-α, pg/ml (mean ± SEM)	62.8±8.8	77.6±6.1	ns
PAF, ng/ml (mean ± SEM)	146.1±11.5	120.9±9.7	ns

*ACPA* anti-citrullinated protein antibodies, *DAS44* Disease Activity Score in 44 joints, *IgM* immunoglobulin M, *IQR* interquartile range, *ns* not significant, *PAF* platelet-activating factor, *RA* rheumatoid arthritis, *RF* rheumatoid factor, *TNF* tumour necrosis factor

### Platelet isolation

Platelets were isolated from fresh buffy coats or from freshly drawn venous blood obtained from healthy control subjects or patients with RA and then collected into citrate-containing blood tubes. Platelets were isolated as described elsewhere [36]. Platelet-rich plasma was prepared by centrifugation (156×*g*, 15 min, 20 °C). Next, a 0.1 vol of acid-citrate-dextrose (2.5 % trisodium citrate, 1.5 % citric acid, 2 % d-glucose) was added to lower the pH of the plasma to 6.5, preventing activation during further isolation. Subsequently, platelets were purified by centrifugation (330×*g*, 15 min, 20 °C), and the platelet pellet was resuspended in HEPES-buffered Tyrode’s solution (145 mM NaCl, 5 mM KCl, 0.5 mM Na_2_HPO_4_, 1 mM MgSO_4_, 10 mM 2-[4-(2-hydroxyethyl)piperazin-1-yl]ethanesulfonic acid [HEPES], pH 6.5) containing 5 mmol/L d-glucose. Prostaglandin I_2_ (Cayman Chemical, Ann Arbor, MI, USA) was added to a final concentration of 10 ng/ml. Next, the washing procedure was repeated, but now the platelet pellet was resuspended in HEPES-buffered Tyrode’s solution, pH 7.2, containing 5 mmol/L d-glucose to a final concentration of 2×10^8^ platelets/ml. Before the experiments, platelets were left at room temperature (RT) for at least 30 minutes to ensure a resting state. Mean platelet volume (MPV), platelet distribution width (PDW) and platelet concentration were determined within 2 h from blood collection to minimise the effect of platelet swelling upon storage (Sysmex KX-21N automated haematology analyser; Sysmex, Kobe, Japan).

### Platelet incubation in the presence of plasma

Platelets were isolated from fresh buffy coats (2×10^8^ platelets/ml) and incubated for 90 minutes at 37 °C in the presence of diluted plasma (final dilution 1:6). Next, platelets were centrifuged (330×*g*, 15 min, 20 °C), and then supernatants were collected and stored at −20 °C until further analysis. Platelet activation was determined by flow cytometry to measure the expression of P-selectin and the presence of platelet aggregates. Furthermore, the production of sCD40L was determined using an enzyme-linked immunosorbent assay (ELISA; eBioscience, San Diego, CA, USA) according to the manufacturer’s protocol. When applicable, change in sCD40L was calculated to correct for the presence of sCD40L in plasma.

### Flow cytometry

To determine P-selectin expression on platelets, platelets were stained with CD62P phycoerythrin (clone AK-4; BD Biosciences, San Jose, CA, USA) diluted in HEPES-buffered Tyrode’s solution, pH 7.2 (1:40 dilution), and incubated for 20 minutes at RT, after which cells were fixed using 1 % paraformaldehyde (no wash) and analysed by flow cytometry (FACSCalibur; BD Biosciences). Analysis was performed using FACSDiva software (BD Biosciences) and FlowJo software (Tree Star, Ashland, OR, USA). Aggregate analysis was based on the appearance of a larger population in the forward scatter (FSC) and side scatter (SSC) after stimulation as described by Matzdorff et al. [37] (Additional file 1: Figure S1). For this experiment, the purity of platelets was determined using CD41 (>95 %), and the position of the platelet aggregate population was determined by activating platelets with 5 μM thrombin receptor-activating peptide. Next, CD62P expression of the aggregated and non-aggregated platelets was determined (Additional file 1: Figure S1a). We observed a nice correlation (Pearson’s *r*=0.5130; *P*<0.0001) between the percentage of aggregates and P-selectin expression, both determined by flow cytometry after incubation in the presence of plasma samples from cohort 1 (control subjects and patients with RA), validating our method of detecting aggregates (Additional file 1: Figure S1b).

### ACPA purification using CCP2-coated beads

ACPA-IgG and ACPA-depleted IgG were purified from plasma of patients with RA by antigen affinity chromatography using cyclic citrullinated peptide 2 (CCP2)-coated beads (Pierce Biotechnology/Thermo Scientific, Rockford, IL, USA). The latter were prepared by mixing 500 μl of biotinylated CCP2 dissolved in phosphate-buffered saline (PBS) at 1 mg/ml with 5 ml of Pierce NeutrAvidin Plus UltraLink slurry resin (Thermo Scientific) for 1 h at RT. Following extensive washing with PBS, the CCP2-coated beads were dissolved in PBS to reach a total volume of 7.5 ml, and 75 μl of bead solution were loaded into each well of a 96-well filter plate (OF 1100; Orochem Technologies, Naperville, IL, USA). Plasma samples (diluted 1:5 in PBS) were applied on beads, and ACPA binding to CCP2 was allowed by vortexing the plate at 600 rpm for 2 h at RT. The plate was then centrifuged for 2 minutes at 500×*g*, and the flow-through was collected in a 96-well deep-well storage plate (Pierce Biotechnology/Thermo Scientific). Beads were then washed sequentially twice with 150 μl of PBS and once with 25 mM ammonium bicarbonate. Of note, the first washing of the beads was combined with the flow-through. ACPA elution was performed by twice adding 150 μl of 100 mM formic acid at pH 2.5 to the beads. The elution fraction was immediately neutralised with 2 M Tris buffer. ACPA-depleted IgG (flow-through) and eluted ACPA-IgG were further analysed by ELISA. ACPA percentages were calculated by setting total IgG (flow-through + elution) at 100 %.

### Measurement of IgG-ACPA

Plasma samples were assessed for the presence of IgG-ACPA by ELISA based on reactivity against CCP2 (Euro Diagnostica, Nijmegen, the Netherlands). Plasma samples were tested at a 1:50 dilution or higher according to the manufacturer’s instructions, using rabbit anti-human IgG-horseradish peroxidase (Dako, Heverlee, Belgium) and 2,2′-azinobis(3-ethylbenzthiazoline-6-sulfonic acid) (Sigma-Aldrich, Zwijndrecht, Belgium).

### ACPA-mediated activation of platelets

Plates were coated overnight with streptavidin (25 μg/ml) and subsequently incubated for 1 h at 37 °C with biotinylated citrulline- and arginine-containing peptides (1 μg/ml). Plates were then incubated with ACPA^pos^ or ACPA^neg^ plasma (1:5 dilution in PBS + 0.1 % bovine serum albumin) at 37 °C for 1 h. After extensive washing of the coated plates, platelets isolated from healthy volunteers were incubated for 2 h at 37 °C, after which supernatants were collected by centrifugation (330×*g*, 15 min, 20 °C) and platelets were resuspended for staining for fluorescence-activated cell sorting analysis. In FcγRIIa blocking experiments, platelets were pre-incubated with anti-CD32 (clone IV.3; STEMCELL Technologies, Vancouver, BC, Canada) or isotype control (mouse IgG2b; eBioscience) for 1 h at 37 °C. ACPA binding to the plates was confirmed by standard ELISA in separate coated wells (data not shown). All experiments were performed in triplicates. The ratio was calculated by dividing the percentage of CD62P^pos^ platelets in the presence of citrulline-containing peptides by the percentage of CD62P^pos^ platelets in the presence of arginine-containing peptides.

### Statistical analysis

Normality testing was performed using the Kolmogorov-Smirnov test (with the Dallal-Wilkinson-Lilliefor correction for *P* value). For normally distributed populations, mean ± standard deviation values are depicted (unless stated otherwise) and Student’s *t* test was used. For non-normally distributed populations, the median and IQR are depicted and significance was determined using the Mann-Whitney *U* test or the corresponding analysis of variance when multiple groups were compared. Correlations were determined using the Pearson’s *r* correlation coefficient, as all populations tested were normally distributed. Linear regression analyses were performed with group (group 1 = control subjects, group 2 = patients with RA) as the independent variable and CD62P aggregates, and sCD40L as dependent variables. To test for paired samples, the Wilcoxon matched pairs test was used. Fisher’s exact test was used to determine the differences between sexes in the two groups. Data were analysed using GraphPad Prism version 5.0 software (GraphPad Software, La Jolla, CA, USA). Linear regression analyses were performed using STATA version 12 software (StataCorp, College Station, TX, USA).

## Results

### Plasma derived factors from patients with RA increase platelet activity

**Fig. 1:**
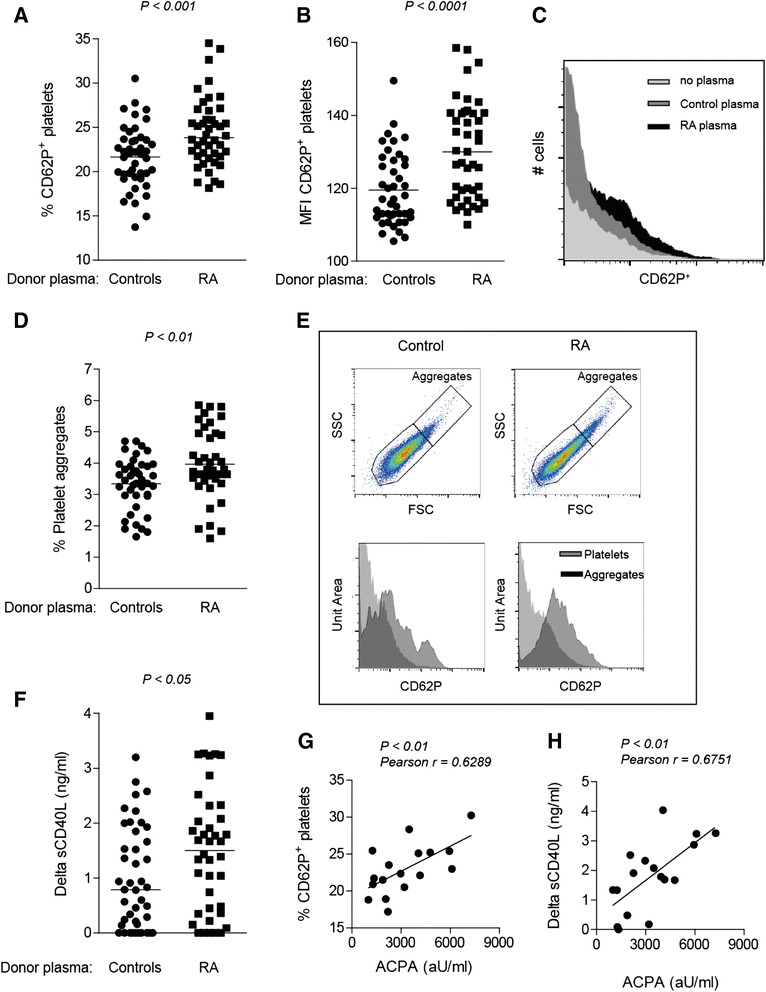
Plasma-derived factors enhance platelet activity. Platelets were isolated from buffy coat and incubated in the presence of plasma obtained from patients with rheumatoid arthritis (RA) or age- and sex-matched healthy controls (cohort 1). Plasma from patients with RA induced higher platelet activation than did plasma from healthy donors, as indicated by (**a**) increased percentage and (**b**) mean fluorescence intensity (MFI) of P-selectin (CD62P) expression, (**d**) platelet aggregation and (**f**) soluble CD40 ligand (sCD40L). **c** Histogram overlay showing representative examples of CD62P expression. **e** Representative pictures of aggregate formation and CD62P expression of non-aggregated and aggregated platelets. **f** Values of sCD40L were corrected for amounts present in plasma. Levels of (**g**) P-selectin and (**h**) sCD40L production correlated with high levels of anti-citrullinated protein antibodies (ACPA; > 1000 AU/ml). Each symbol represents a plasma sample. *FSC* forward scatter, *SSC* side scatter

As described, patients with RA display a more activated platelet phenotype, but the underlying mechanism is not clear. To investigate if plasma-derived factors could have contributed to this observation, we incubated freshly isolated platelets from healthy volunteers with plasma obtained from patients with RA. To ensure moderate to high disease activity, only plasma of patients with a DAS44 > 2.4 were included. Plasma from age- and sex-matched healthy volunteers was used as a control (cohort 1). Increased expression of P-selectin (21.6±0.5 % vs 24.4±0.6 %; *P*<0.001) (Fig. 1a), increased P-selectin mean fluorescence intensity (MFI) (119.5±1.5 vs 130.5±2.0; *P*<0.0001) (Fig. 1b), more platelet aggregation (3.3±0.1 vs. 3.9±0.2; *P*<0.01) (Fig. 1d) and enhanced production of sCD40L (0.99±0.0.14 ng/ml vs 1.5±0.18 ng/ml; *P*<0.05) (Fig. 1f) were detected after incubation of platelets of healthy subjects in the presence of plasma from patients with RA. Notably, linear regression analysis indicated that platelet characteristics such as P-selectin expression, aggregate formation and the production of sCD40L induced by a given plasma sample predicted group in the plasma donor (control vs patients with RA) (Table 2).

**Table 2:** Linear regression analysis

Dependent variable	β-coefficient	SEM	*t*	*P* value	β	95 % CI	
CD62P	2.01	0.80	2.50	0.01	0.27	0.41	3.60
Aggregates	0.47	0.24	1.96	0.05	0.21	−0.01	0.95
sCD40L	0.51	0.23	2.24	0.03	0.24	0.06	0.96

*CD62P* P-selectin, *CI* confidence interval, *sCD40L* soluble CD40 ligand, *SEM* standard error of the mean

The β-coefficient represents the effect sizes from the regression analyses of the association between platelet characteristics induced by a given plasma sample and group status of the plasma donor. β-values represent the standardised regression coefficients

The *t* statistic (*t*) is the beta-coefficient divided by its standard error

Next, we studied which factors in plasma could be responsible for the observed platelet activity. Platelet-activating factor (PAF) is a potent inflammatory phospholipid implicated in the release of various cytokines involved in RA, such as tumour necrosis factor (TNF)-α, IL-1 and IL-6. In addition, TNF-α is able to activate platelets through stimulation of the arachidonic acid pathway [38]. Therefore, we determined TNF-α and PAF levels in the plasma samples used. We did observe higher TNF-α levels in the plasma of patients with RA (median 87.8 pg/ml, IQR 57.8–126.4)than in controls (median 75.0 pg/ml, IQR 51.4–88.9) (*P*<0.05) and increased levels of PAF (RA 33.4±1.7 ng/ml vs controls 27.9±1.4 ng/ml; *P*<0.05) (data not shown). However, we could not find a correlation between TNF-α and/or PAF with platelet characteristics (Additional file 2: Figure S2g-h) or disease activity (data not shown).

Because platelets express FcγRIIa, we next questioned if ACPA could potentially mediate the activation of platelets. Although we could not detect a correlation between ACPA and P-selectin expression on platelets in all patients with RA (Additional file 2: Figure S2a), we did observe a strong correlation between patients with higher ACPA levels (>1000 AU/ml) and the expression of both P-selectin (Fig. 1g) (Pearson’s *r*=0.6289, *P*<0.01) and the production of sCD40L by platelets (Fig. 1h) (Pearson’s *r*=0.6751, *P*<0.01). No correlation was observed between platelet characteristics and RF-IgM (Additional file 2: Figure S2e,f). These data indicate that platelet activation induced by a given plasma sample predicts group of the plasma donor (control vs patients with RA) and points towards an association between platelet activation and ACPA level.

### ACPA-mediated activation of platelets

**Fig. 2:**
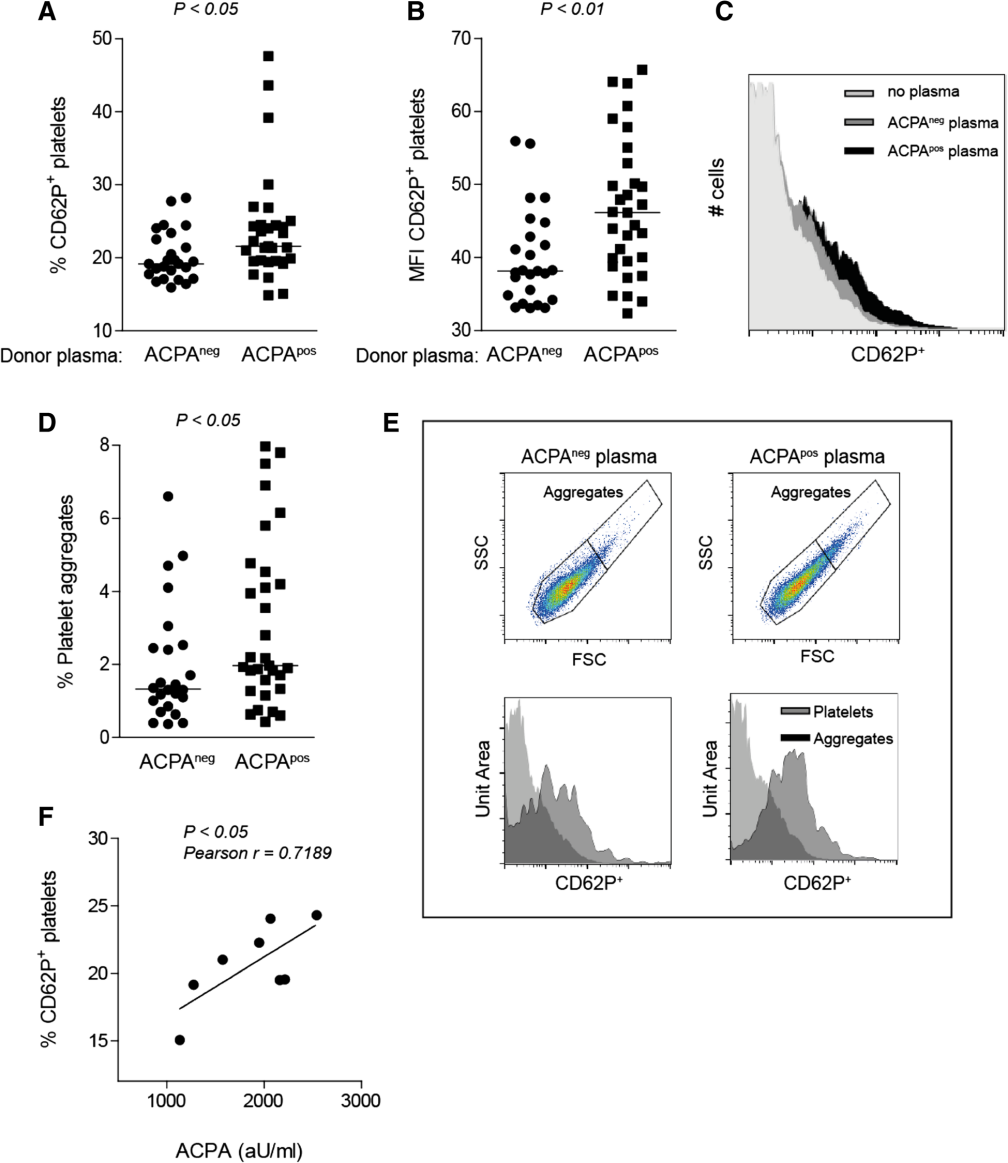
ACPA-mediated activation of platelets from healthy subjects. Platelets were isolated from buffy coat and incubated in the presence of plasma obtained from ACPA^neg^ or ACPA^pos^ patients with RA (matched for age, sex and DAS44; cohort 2). To ensure we were studying active disease, only plasma samples from patients with a DAS44 > 2.4 were included. Plasma from ACPA^pos^ patients with RA induced higher platelet activation than did plasma from ACPA^neg^ patients with RA, as indicated by increased (**a**) percentage and (**b**) MFI of CD62P expression and (**d**) platelet aggregation. **c** Histogram overlay showing representative examples of CD62P expression. **e** Representative pictures of aggregate formation and CD62P expression of non-aggregated and aggregated platelets. **f** Levels of P-selectin correlated with high ACPA titres (> 1000 AU/ml). Each symbol represents a plasma sample. *ACPA* anti-citrullinated protein antibodies, *CD62P* P-selectin, *DAS44* Disease Activity Score in 44 joints, *FSC* forward scatter, *MFI* mean fluorescence intensity, *RA* rheumatoid arthritis, *SSC* side scatter

**Fig. 3:**
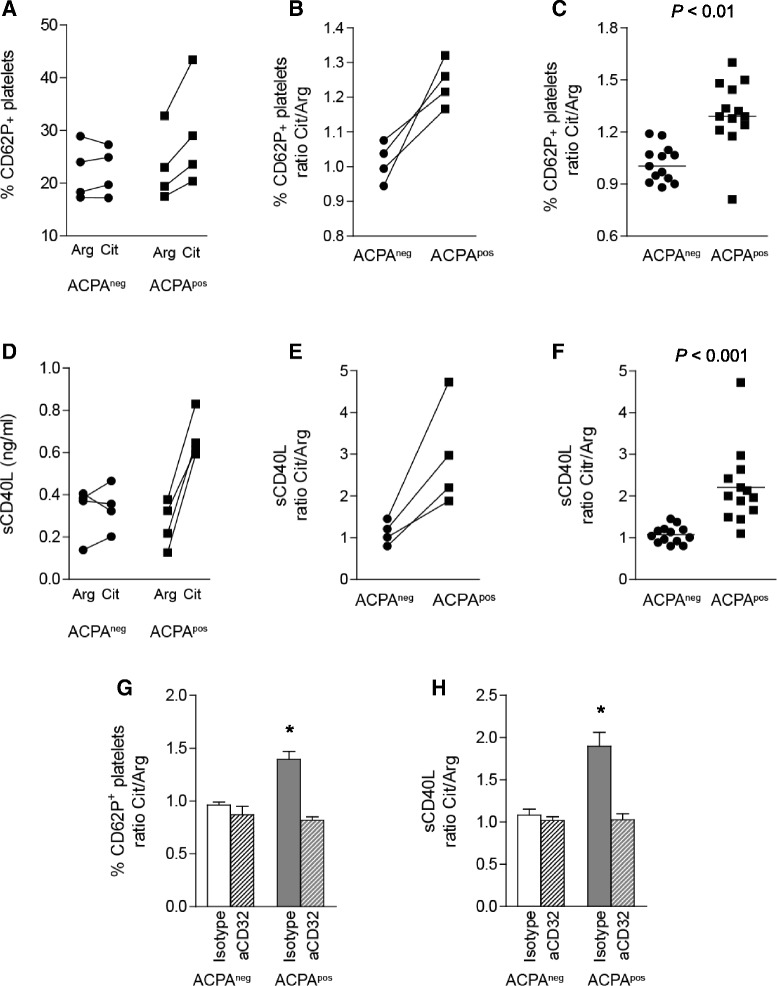
Citrulline-specific activation of platelets is FcγRIIa-dependent. Platelets were isolated from healthy donors. ACPA plate-bound immune complexes were generated by incubating ACPA^neg^ and ACPA^pos^ plasma on wells coated with citrulline- or arginine-containing peptides. Representative examples of citrulline-specific increase in P-selectin expression and sCD40L release are shown in **a** and **d**, respectively. **b** and **e** These effects are still present after correcting for aspecific binding by calculating the citrulline/arginine ratio. **c** and **f** Summaries of three independent experiments. Pre-incubation of platelets with a blocking antibody directed against FcγRIIa abolished the citrulline-specific induction of P-selectin (**g**) and sCD40L release (**h**). *ACPA* anti-citrullinated protein antibodies, *CD62P* P-selectin, *FcγRIIa* low-affinity immunoglobulin G receptor, *sCD40L* soluble CD40 ligand. *P < 0.05

To further investigate the relationship between ACPA level and platelet activation, we incubated platelets from healthy subjects with plasma from either ACPA^neg^ or ACPA^pos^ patients with RA (cohort 2; age-, sex- and DAS44-matched). We again observed platelet activation when plasma from ACPA^pos^ patients was used as indicated by increased platelet P-selectin expression (Fig. 2a) (ACPA^neg^ median 19.2, IQR 17.4–23.0; ACPA^pos^ median 21.6, IQR 19.5–24.5; *P*<0.05), increased CD62P MFI (Fig. 2b) (ACPA^neg^ median 38.1, IQR 34.5–43.8; ACPA^pos^ median 46.2, IQR 39.5–52.9; *P*<0.01) and increased number of platelet aggregates (Fig. 2d) (ACPA^neg^ median 1.3, IQR 0.9–2.5; ACPA^pos^ median 1.97, IQR 1.3–4.5; *P*<0.05). Like the observations in cohort 1, a strong positive correlation was again seen between plasma samples with high ACPA level and its ability to induce P-selectin expression on platelets from healthy subjects (Pearson’s *r*=0.7189, *P*<0.05) (Fig. 2f). To address the question whether ACPA could directly activate platelets, we coated plates with arginine- or citrulline-containing peptides and incubated the coated plates with plasma from RF^neg^ACPA^pos^ patients to generate platelet-bound ACPA–immune complexes. Plasma from RF^neg^ACPA^neg^ patients was used as a negative control. We observed a citrulline-dependent activation of platelets because neither the arginine control nor the use of ACPA^neg^ plasma resulted in increased P-selectin expression or sCD40L release (Fig. 3a–f). The ACPA-mediated activation of platelets was FcγRIIa-dependent because pre-incubation of platelets with anti-CD32 inhibited upregulation of P-selectin expression and sCD40L release (Fig. 3g,h).

### Enhanced platelet activation correlates with disease activity

**Fig. 4:**
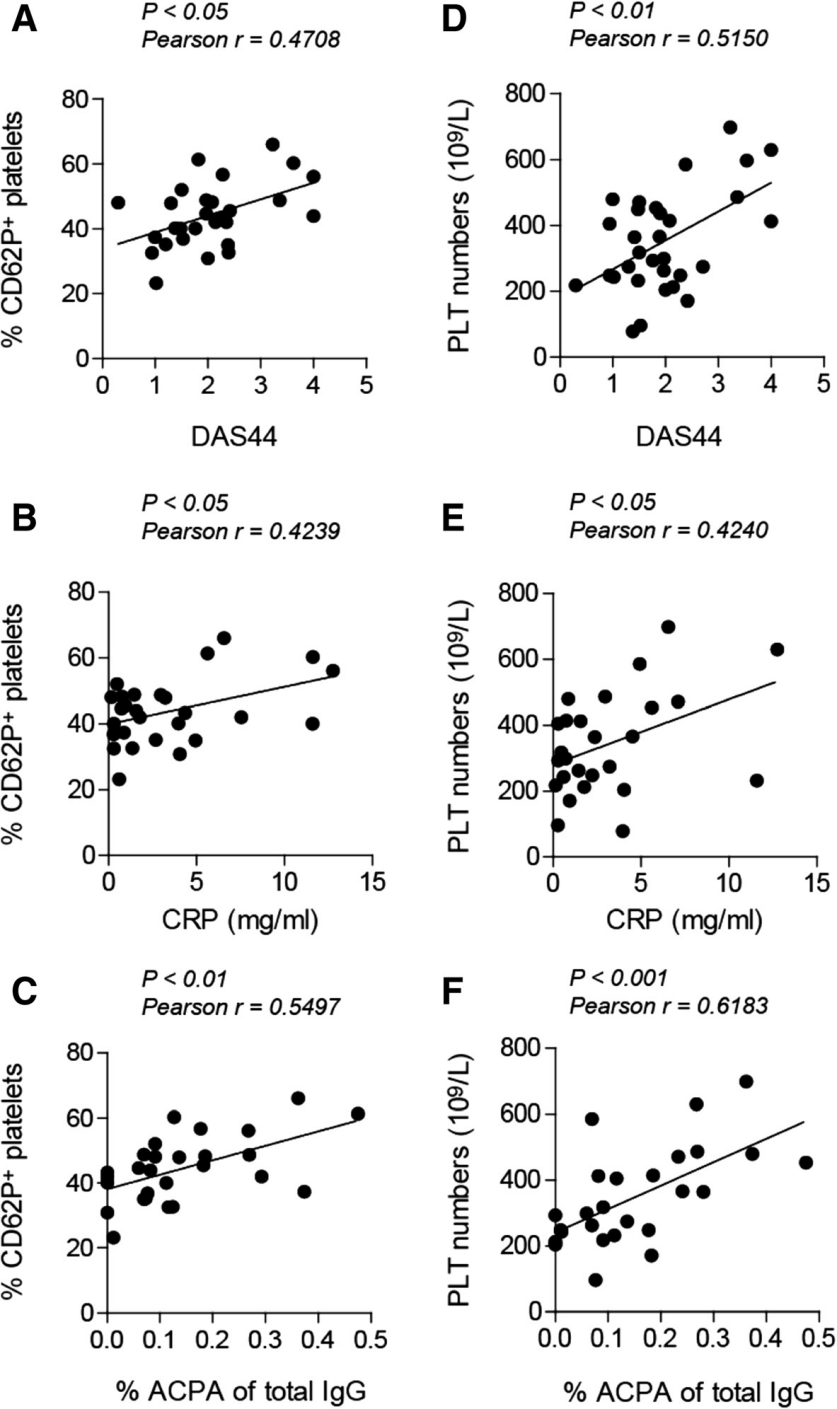
Platelet phenotype correlates with disease activity. The expression of P-selectin (CD62P) was determined on isolated platelets from patients with rheumatoid arthritis (RA) and correlated with Disease Activity Score in 44 joints (DAS44) (**a**), C-reactive protein (CRP) levels (**b**) and anti-citrullinated protein antibodies (ACPA) (**c**). Platelet (PLT) numbers correlated with DAS44 score (**d**), CRP levels (**e**) and ACPA levels (**f**). *IgG* immunoglobulin G

In addition to increased P-selectin expression and sCD40L release, platelets transform from a normal discoid shape into a more spherical shape with protrusions of pseudopods during activation. This is visualised as increased MPV and enhanced PDW. As plasma from patients with RA is capable of inducing platelet activation, we next compared freshly isolated platelets from patients with RA with those of healthy controls. We observed a higher basal activation status of platelets isolated from patients with RA, as indicated by increased MPV, PDW, platelet numbers and expression of P-selectin (Table 3). Because there is variation in total IgG levels between patients, we corrected for the presence of IgG by isolating ACPA and calculating the percentage of ACPA to total IgG. The expression of P-selectin on platelets from patients with RA correlated with DAS44 (Pearson’s *r*=0.4708, *P*<0.05), C-reactive protein (CRP) levels (Pearson’s *r*=0.4239, *P*<0.05) and the percentage of ACPA (Pearson’s *r*=0.5497, *P*<0.01) (Fig. 4a–c). In addition, the number of platelets also correlated with DAS44 (Pearson’s *r*=0.5150, *P*<0.01), CRP (Pearson’s *r*=0.4240, *P*<0.05) and percentage of ACPA (Pearson’s *r*=0.6183, *P*<0.001) (Fig. 4d–f). Together, these findings indicate enhanced platelet activation in patients with RA which correlates with disease activity and the presence of ACPA.

**Table 3:** Characteristics isolated platelets from healthy controls vs patients with RA

	Healthy controls	Patients with RA	*P* value
Total number	29	32	
Female, n (%)	16 (55.2)	22 (68.75)	
Male, n (%)	13 (44.8)	10 (31.25)	
Age, yr	37.4±1.79	63.5±2.24	< 0.0001
Mean platelet volume (fl)	7.88±0.137	8.41±0.183	0.0273
Platelet distribution width (fl)	9.71±0.217	10.13±0.295	ns
Platelet numbers (10^9^/L)	298.3±21.29	383.9±28.66	0.0187
P-selectin expression (%)	17.94±1.188	44.28±1.889	< 0.0001

*ns* not significant

## Discussion

Besides their well-established role in haemostasis and thrombin generation, ample data are now emerging on the immunoregulatory functions of platelets [1–3]. Platelets are closely involved in the initiation and/or perpetuation of inflammatory events and cellular influx taking place at the endothelium–synovium interface by releasing a large plethora of cytokines, chemokines and growth factors. Furthermore, recent data tend to support a relatively unexplored role for platelets during the inflammatory events of RA [13]. Initially, the involvement of platelets in joint inflammation was described in mice. Platelets were found to interact with and adhere to endothelial cells and leucocytes in the inflamed synovial vessels and also to contribute to the production of arthritogenic prostaglandins in the K/BxN mouse model [39–41]. Furthermore, platelet depletion reduced vascular leakage in arthritic joints and attenuated inflammatory arthritis [4, 11, 12]. Now, data are emerging on platelet activation in patients with RA [15–19, 42]. Studying the phenotype of freshly isolated platelets from patients with RA, we indeed observed an increased activation status, as indicated by the MPV, platelet numbers and P-selectin expression of patients with RA compared with healthy controls. MPV increase during aging, which is still debated, could represent a limitation of these findings, as these groups were not matched for age [43, 44]. However, among the patients with RA, we observed a correlation between P-selectin expression and DAS44, CRP and the percentage ACPA to total IgG, confirming and extending the correlation between activation of platelets and disease activity [45]. Treatment of patients with RA included a wide variety of DMARDs, biologic agents and glucocorticoids. Over time, several patients received different forms of medication or even a combination of drugs. The effect of anti-arthritis medications on blood-related prothrombotic and proinflammatory parameters has been reviewed by Beinsberger et al. [46], who compared conventional therapy with methotrexate and anti-TNF treatment and showed that anti-arthritis medications have a normalising effect on blood-related prothrombotic and proinflammatory parameters. Furthermore, sulphasalazine inhibits arachidonic acid–induced platelet aggregation similarly to the way aspirin and hydroxychloroquine reverse platelet activation induced by human IgG antiphospholipid antibodies [47, 48]. Together, these data indicate that anti-rheumatic treatment actually reduces platelet activation and therefore is unlikely to have contributed to the increased platelet activation observed in our study.

Previous studies indicated that platelets from patients with RA express higher P-selectin levels and produce higher amounts of sCD40L [15, 42]. In the present study, we show that incubation of platelets from healthy subjects with plasma derived from patients with RA induces an activated platelet phenotype, as indicated by elevated P-selectin expression, increased sCD40L production and enhanced aggregation. This indicates that RA plasma-derived factors can induce platelet activation. PAF and TNF-α are important mediators in RA that are known to be involved in the activation of platelets [38, 49, 50]. We indeed observed elevated levels of both PAF and TNF-α in plasma of patients with RA, but we could not find a correlation with platelet characteristics. As platelets express only FcγRIIA, which is known for binding IgG immune complexes, we hypothesised that the presence of immune complexes containing autoantibodies could mediate platelet activity [51]. Interestingly, in platelet aggregometry studies done many years ago, researchers identified IgG-containing immune complexes as potential triggers for excessive platelet aggregation [52, 53]. Furthermore, IgG immune complexes collected from the serum of patients with systemic lupus erythematosus were shown to activate healthy platelets and that blocking the FcγRIIa receptor or depleting the serum of immunoglobulins abolished the increased platelet activation [54]. Moreover, exposure to complexed IgG increases the sensitivity towards low doses of thrombin, which could result in accelerated vascular complications in vivo [55]. Our studies show the involvement of FcγRIIA in mediating platelet activation in the presence ACPA.

We describe a strong correlation between high ACPA levels and platelet characteristics and show that platelets can be activated by plate-bound ACPA. Recently, it became apparent that human platelets do not express the IgM Fc receptor, making it unlikely that platelets become activated in the presence of IgM-containing immune complexes [56]. Indeed, in our present study, we did not observe a correlation between RF-IgM and platelet activation. Notably, these data are in accordance with the observations of Scott et al., who found no correlation between platelet aggregation and IgM-RF or IgG-RF in patients with RA [57].

ACPA-mediated activation of platelets is potentially of relevance for the earliest phases of RA because, during vascular injury, a tripartite interaction between the damaged endothelium, platelets and neutrophils develops. Wipke et al. elegantly showed that, within minutes of intravenous injection, autoantibodies specifically localise to joints destined to become inflamed in the K/BxN mouse model of RA, even in the absence of pre-existing joint inflammation [58]. Furthermore, the formation of immune complexes on the articular surface initiates an inflammatory cascade leading to increased vascular permeability [59]. The specific homing of autoantibodies to the joints could contribute to local activation of platelets, which in turn release important mediators of vascular permeability, such as IL-1 and serotonin [4, 11]. Importantly, patients with RA have an increased mortality risk (50 % higher) relative to the healthy population, which is largely attributable to increased cardiovascular disease (CVD), particularly atherosclerosis. As endothelial dysfunction is the initiating step in atherosclerosis, and because of the prominent role of platelets during CVD, it could well be that ACPA-mediated activation of platelets contributes to CVD in patients with RA. Indeed, seropositivity for RF and ACPA is associated with increased CVD risk in patients with RA and is related to impaired endothelial function [30–32, 34]. Remarkably, Cambridge et al. recently described an association between anti-CCP antibodies and coronary heart disease even in the absence of rheumatic disease [26].

## Conclusions

Although several factors are at play in influencing platelets during established RA, we believe that, especially in the initiating (symptom-free) phase of the disease, ACPA-mediated activation of platelets could have an important role in enhancing the basal activation status of platelets, as ACPA can be detected many years before disease onset. Because platelets carry various mediators facilitating the recruitment of circulating monocytes and/or leucocytes to injured endothelium and have the potential to propagate vascular permeability, this ACPA-mediated activation of platelets could be the initialising step of the enhanced erosive damage observed in ACPA^pos^ patients and/or the increased cardiovascular risk. Unfortunately, we had no access to a prospective cohort in which the platelet phenotype of healthy blood donors who in time develop RA was monitored. To confirm our hypothesis, it would be of great interest to investigate whether the occurrence of ACPA coincides with platelet activation in patients with clinically silent RA.

## Additional files

Additional file 1: Figure S1. Visualisation of thrombin receptor-activating peptide (TRAP)-mediated platelet aggregation using flow cytometry. Platelets from healthy donors were isolated and incubated in the presence of TRAP (0, 0.5 or 5 μM TRAP), and the formation of platelet aggregates was analysed. Histograms showing the P-selectin upregulation upon TRAP activation for non-aggregated platelets (*filled histograms*) and aggregated platelets (*open histograms*) (**a**). Correlation between percentage aggregates measured by flow cytometry and P-selectin expression after activation in the presence of all plasma samples of cohort 1. Each symbol represents a plasma sample (**b**). (PDF 78 kb) Additional file 2: Figure S2. Correlations between platelet activation and markers of inflammation. Correlation between percentage P-selectin expression in all patients (**a**) and patients with ACPA > 1000 AU/ml (**b**). Correlation between sCD40L in all patients (**c**) and patients with ACPA > 1000 AU/ml (**d**). Correlation between percentage P-selectin expression and RF-IgM in all patients (**e**) and patients with RF-IgM > 50 IU/ml (**f**). Correlation between TNF-α and P-selectin expression in all patients (**g**). Correlation between PAF and P-selectin expression in all patients (**h**). Each symbol represents a plasma sample. (PDF 67 kb)

## Abbreviations

ACPA: Anti-citrullinated protein antibodies
CCP: Cyclic citrullinated peptide
CD62P: P-selectin
CI: Confidence interval
CRP: C-reactive protein
CVD: Cardiovascular disease
DAS: Disease Activity Score
DMARD: Disease-modifying antirheumatic drug
ELISA: Enzyme-linked immunosorbent assay
FcγRIIa: Low-affinity immunoglobulin G receptor
FSC: Forward scatter
HEPES: 2-[4-(2-hydroxyethyl)piperazin-1-yl]ethanesulfonic acid
IgG: Immunoglobulin G
IL: Interleukin
IQR: Interquartile range
MFI: Mean fluorescence intensity
MPV: Mean platelet volume
PAF: Platelet-activating factor
PBS: Phosphate-buffered saline
PDW: Platelet distribution width
PLT: Platelets
RA: Rheumatoid arthritis
RF: Rheumatoid factor
RT: Room temperature
sCD40L: Soluble CD40 ligand
SEM: Standard error of the mean
SSC: Side scatter
TNF: Tumour necrosis factor
