# Supplementary figures and images for: Anti-citrullinated protein antibodies contribute to platelet activation in rheumatoid arthritis
Source: Arthritis Res Ther. 2015 Aug 24;17(1):209. doi: 10.1186/s13075-015-0665-7 (PMC4548712; doi:10.1186/s13075-015-0665-7)

**A**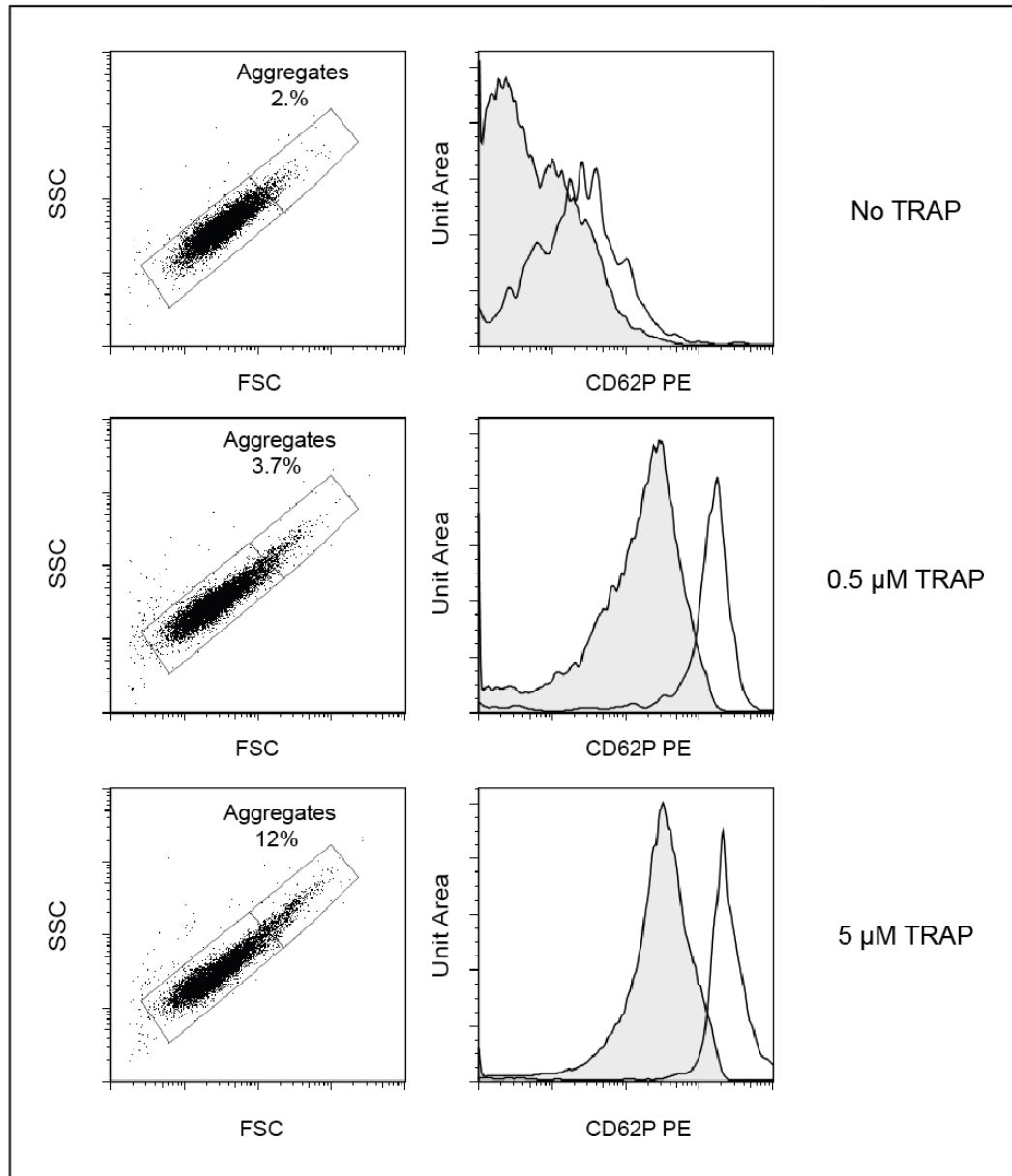**B**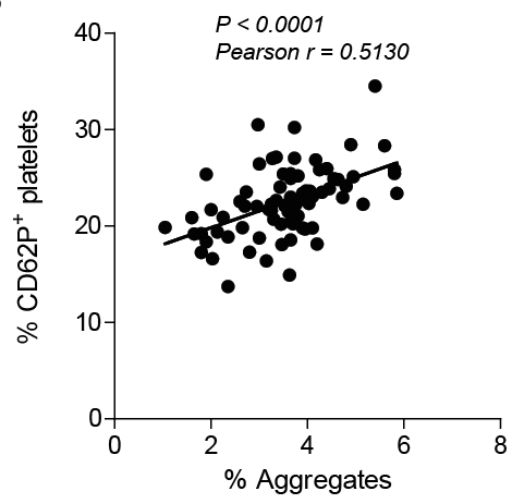**Online Figure 1**

Supplement: Additional file 1: Figure S1. — Visualisation of thrombin receptor-activating peptide (TRAP)-mediated platelet aggregation using flow cytometry. Platelets from healthy donors were isolated and incubated in the presence of TRAP (0, 0.5 or 5 μM TRAP), and the formation of platelet aggregates was analysed. Histograms showing the P-selectin upregulation upon TRAP activation for non-aggregated platelets (filled histograms) and aggregated platelets (open histograms) (a). Correlation between percentage aggregates measured by flow cytometry and P-selectin expression after activation in the presence of all plasma samples of cohort 1. Each symbol represents a plasma sample (b). (PDF 78 kb) [file 13075_2015_665_MOESM1_ESM.pdf]

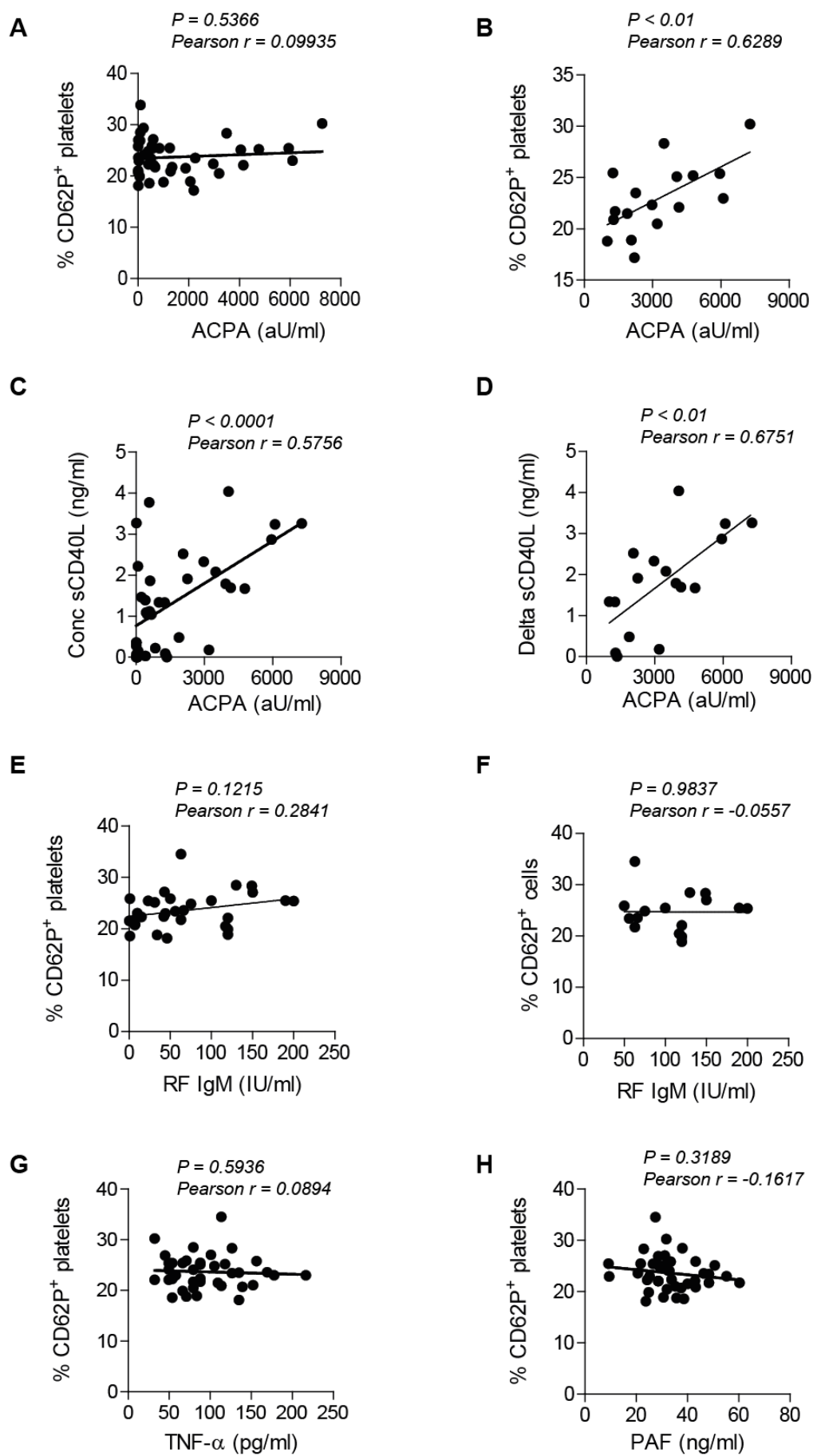

Online Figure 2

Supplement: Additional file 2: Figure S2. — Correlations between platelet activation and markers of inflammation. Correlation between percentage P-selectin expression in all patients (a) and patients with ACPA > 1000 AU/ml (b). Correlation between sCD40L in all patients (c) and patients with ACPA > 1000 AU/ml (d). Correlation between percentage P-selectin expression and RF-IgM in all patients (e) and patients with RF-IgM > 50 IU/ml (f). Correlation between TNF-α and P-selectin expression in all patients (g). Correlation between PAF and P-selectin expression in all patients (h). Each symbol represents a plasma sample. (PDF 67 kb) [file 13075_2015_665_MOESM2_ESM.pdf]
